# PDLIM3 expression is implicated in the invasive behavior of glioblastoma stem cells and their ability to form neurospheres

**DOI:** 10.17912/micropub.biology.001613

**Published:** 2025-07-08

**Authors:** Yvan Nicaise, Caroline Delmas, Elisabeth Cohen-Jonathan-Moyal, Catherine Seva

**Affiliations:** 1 Centre de Recherche en Cancérologie de Toulouse, Toulouse, Occitanie, France; 2 Institut universitaire du cancer de Toulouse Oncopole, Toulouse, Occitanie, France

## Abstract

The recurrence of glioblastoma can be attributed to the high invasiveness and resistance of glioblastoma stem cells (GSCs). Therefore, it is crucial to investigate the molecular mechanisms that drive the agressiveness of these cells. This research focuses on the actin-associated protein PDLIM3 that we found overexpressed in GBM and in highly invasive GSCs. Suppressing PDLIM3 expression with targeted siRNAs significantly decreases cell invasion. Additionally, our findings highlight that PDLIM3 is essential for GSCs in forming neurospheres. This research provides the first evidence that PDLIM3 may contribute to the aggressiveness of glioblastoma by facilitating GSCs sphere formation and enhancing their invasiveness.

**Figure 1. PDLIM3 expression in GBM and GSCs, involvement in the invasive behavior of GSCs and their ability to form neurospheres:**
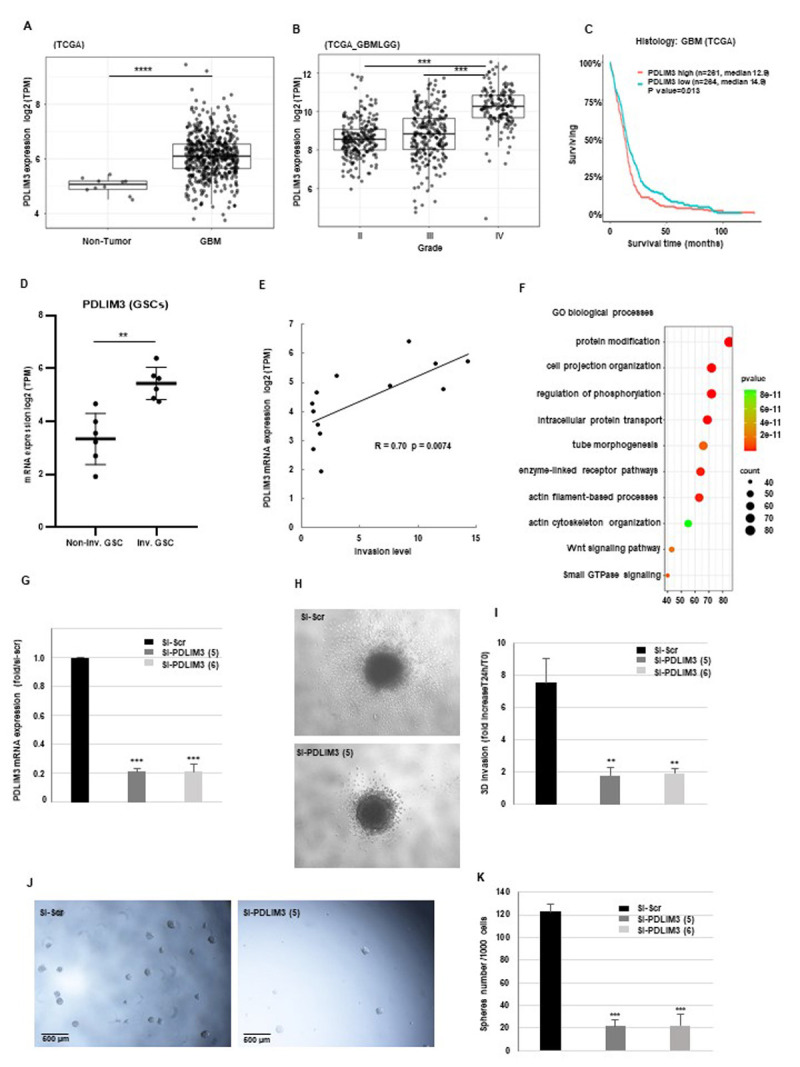
(A, B) The expression levels of PDLIM3 mRNA in non-tumor, low-grade glioma and glioblastoma (GBM) were analyzed using data from the TCGA database. The results are expressed as log2 TPM (Transcripts Per Million). (C) Survival analysis using the Kaplan-Meier estimator was conducted to evaluate the overall survival probabilities of patients with high versus low PDLIM3 expression. (D) PDLIM3 mRNA levels in glioma stem cells (GSCs, n = 13) were examined through RNA sequencing, distinguishing between non-invasive (Non-inv. GSC) and invasive GSCs (Inv. GSC). Results are reported as log2 TPM. (E) The correlation between PDLIM3 expression and GSCs invasion was assessed. (F) Gene ontology analysis was conducted on genes that were significantly upregulated in GSCs with high PDLIM3 expression, as detailed in the "Methods" section. (G) GSCs underwent transfection with specific PDLIM3 siRNAs (si-PDLIM3(5), si-PDLIM3(6)) or a scrambled control (si-Scr), and the mRNA levels of PDLIM3 were measured using real-time PCR, with GAPDH serving as the reference gene for normalization. (H, I) invasive GSCs were transfected with PDLIM3 siRNAs or a scrambled control and subsequently evaluated in a 3D invasion assay, as described in the "Methods." (H) Images depicting the invasion capabilities of GSCs are representative of three independent experiments. (J, K) GSCs were transfected with specific PDLIM3 siRNAs or a scrambled control, and the number of neurospheres was counted microscopically, with representative micrographs taken at a magnification of x20. (K) Quantitative data from three independent experiments are presented as means ± SD, and statistical analyses were performed as described in the "Methods."

## Description

The median overall survival of glioblastoma (GBM), an aggressive tumor of the central nervous system, is less than two years. Its infiltrative nature makes surgical resection difficult and often incomplete. Furthermore, the majority of cases relapse even after receiving post-surgical chemo-radiotherapy (Weller et al. 2017). The presence of GBM stem cells (GSCs), which are very resistant to treatment and have a high potential to invade the surrounding normal brain, is an important cause of the systematic recurrence of glioblastoma (Bao et al. 2006, Ortensi et al. 2013). There are currently no clinical treatments specifically targeting GSCs and the processes governing the invasive potential of GSCs remain poorly understood. Therefore, in order to identify new effective treatments against GBM, it is imperative to understand the molecular mechanisms underlying the invasiveness and resistance of GSCs.

In the present paper, we focused on the PDZ and LIM Domain 3 (PDLIM3) protein which belongs to the large PDLIM family of actin-associated proteins, containing an N-terminal PDZ domain and a C-terminal LIM domain, which are involved in many cell signaling pathways (Jiang et al. 2023). In physiological conditions, PDLIM3 is highly expressed in cardiac and skeletal muscle. It is essential for contractile function and actin cytoskeleton organization via its interaction with α-actinin. It has also an important role in skeletal muscle differentiation (Pomies et al. 1999, Lorenzen-Schmidt et al. 2005, McCord et al. 2011, Ohsawa et al. 2011). PDLIM3 has been little studied in cancer. Elevated expression of PDLIM3 has been reported in pediatric patients with rhabdomyosarcoma, a skeletal muscle tumor (Lak et al. 2021). In gastric, tongue, and bladder carcinoma, PDLIM3 is also overexpressed compared to corresponding healthy tissues and could be a predictive factor for tumor progression and survival of patients with these cancers (Feng et al. 2020, Lee et al. 2021, Hu et al. 2022). Patients with medulloblastoma also have elevated levels of PDLIM3 associated with tumor growth, an increased cell proliferation, and the activation of the Hedgehog signaling pathway (Shou et al. 2015, Zhang et al. 2023).

Furthermore, PDLIM3 has been shown to be associated with the immune response in cancer. Its overexpression was correlated with immune infiltration and poor prognosis in gastric carcinomas (Hu et al. 2022). A role of PDLIM3 has also been reported in tumorigenesis and metastatic potential of pancreatic cancer, with a decrease in the migratory properties of pancreatic cancer cells when its expression is downregulated (Lakshmanan et al. 2022). The expression and role of PDLIM3 in GBM and GSCs in particular has never been reported in the literature.

Here, we compared the PDLIM3 mRNA expression levels between non-tumor brain samples and GBM samples from TCGA and Rembrandt databases. Figures 1A and extended data 1A show that the expression of PDLIM3 was significantly higher in GBM samples compared with non-tumor brain samples in both databases. We subsequently assessed the relationship between PDLIM3 expression and the aggressiveness of tumors. To achieve this, we examined PDLIM3 expression in relation to tumor grade. The findings from 3 different databases TCGA-GBMLGG (figure 1B) and Rembrandt (extended data 1B) and CGGA (extended data 1C) indicate a significantly higher PDLIM3 expression in the most aggressive grade IV GBMs. In order to evaluate the prognostic significance of PDLIM3 in glioblastoma (GBM), we created Kaplan-Meier survival analysis curves utilizing PDLIM3 expression data sourced from the different databases. Our findings revealed that elevated levels of PDLIM3 expression were significantly linked to reduced survival rates among GBM patients of the TCGA and CGGA databases (figures 1C and extended data 1D). The results from the Rembrandt database presented in extended data 1E indicate that a high level of PDLIM3 expression is also associated with predictive value regarding survival in patients with astrocytoma.

We previously evaluated the invasive capabilities of GSCs derived from 13 human GBM biopsies utilized in this study through an in vitro 3D invasion assay (Lacore et al. 2022).The extent of the area occupied by invading cells was assessed 24 hours after their incorporation into Matrigel, as described in the “Methods” section. GSCs were classified into two groups: highly invasive and low invasive, determined by a neurosphere surface area/invading cell surface area ratio greater than 2. We have also previously conducted RNA sequencing on GSCs obtained from the 13 human GBM biopsy samples. Analysis of PDLIM3 expression through RNA sequencing revealed a markedly elevated level in the highly invasive group of GSCs when compared to the low invasive group (figure 1D). The correlation curve, derived from the expression data of PDLIM3 and the invasion levels in the 13 GSC samples, validated these results (figure 1E). We utilized the RNA sequencing data to identify genes that are significantly up-regulated in GSCs exhibiting high levels of PDLIM3 expression, in comparison to those with low PDLIM3 expression. By applying a threshold of a ≥1.7-fold change and a p-value of <0.05, we identified 1302 up-regulated genes. The complete list of these genes can be found in Supplementary Table S1. Subsequently, we conducted a pathway enrichment analysis on the 1302 up-regulated genes associated with GSCs that have elevated PDLIM3 expression. The results, presented in figure 1F, suggest a possible role of PDLIM3 in enhancing the invasive properties of GSCs, as several enriched pathways, such as cell projection organization, actin filament organization, and cytoskeleton organization, are linked to migration and invasion mechanisms. In particular, several proteins common to these 3 pathways, including vimentin, a constituent of intermediate filaments, DBN1, an actin-binding protein, or GPM6A, a membrane glycoprotein linked to the cytoskeleton, show an expression strongly and significantly correlated with that of PDLIM3 (extended data 1E-G). These proteins contribute to the assembly and reorganization of actin, the stabilization of the cytoskeleton and the formation of filopodia. Their important role in the invasion of tumor cells and GBM cells in particular is established in the literature (Terakawa et al. 2013, Zottel et al. 2021, Lacore et al. 2022). PDLIM3, which is part of the actin-binding protein family, could play a role in invasion by contributing to the organization of this network. The reorganization of the actin cytoskeleton is particularly crucial for the migratory behavior of cells, including in the context of gliomas. To investigate the potential role of PDLIM3 in the invasive properties of GSCs, we performed 3D invasion assays utilizing invasive GSCs neurospheres obtained from GBM biopsy samples, in which PDLIM3 was knocked down using a specific siRNA si-PDLIM3(5), validated for its efficiency to inhibit its expression in the GBM neurospheres compared to a scramble control (figure 1G). As depicted in figures 1H-I, GSCs spheroids deficient for PDLIM3 exhibited a marked reduction in invasive capability compared to those transfected with a scrambled control, thereby confirming the involvement of this factor in the invasion of GSCs. Additionally, figure 1H illustrates the diminished invasion capacity of spheroids treated with an anti-PDLIM3 siRNA in the 3D invasion assay. To assess a potential off-target effect of the PDLIM3 siRNA, we used a second siRNA, si-PDLIM3(6) and showed in GBM neurospheres, a high inhibition of PDLIM3 expression (figure 1G) as well as similar results in 3D invasion assays (figure 1I).

Finally, we also investigated the impact of PDLIM3 on the sphere-forming capacity of GSCs. The formation of neurospheres was assessed in GSCs that were transfected with two distinct PDLIM3 siRNAs or a scrambled control. Our findings revealed a notable reduction in the number of spheres when PDLIM3 expression was inhibited (figures 1J, K). Figures 1K presents representative photographic images that demonstrate a reduction in the quantity of neurospheres generated by GSCs upon the inhibition of PDLIM3 expression.

This study is the first to demonstrate that PDLIM3 is expressed in GBM cells and GSCs and may contribute to GBM tumorigenicity and aggressiveness by promoting GSCs sphere formation and increasing their invasive abilities.

## Methods

***Culture of GSCs derived from GBM biopsy specimens.*** Biopsies of glioblastoma (GBM) were collected from the Department of Neurosurgery at Toulouse University Hospital. This clinical investigation, directed by Professor E. Cohen-Jonathan-Moyal, received authorization from the Human Research Ethics Committee (Ethics Code 12TETE01, ID-RCB No. 2012-A00585-38, Approval Date: May 7, 2012). All participants provided written informed consent. The primary neurospheres of glioblastoma stem cells (GSCs) were derived from GBM samples using the technique outlined by Avril and were cultured in DMEM-F12 (GIBCO) enriched with B27 and N2 (Life-Technologies), along with FGF-2 and EGF (Peprotech). The GSCs utilized in this research have been previously characterized, demonstrating self-renewal capabilities, overexpression of stem cell markers, the potential to differentiate into neural lineages, and the ability to form tumors in vivo.

***Bioinformatic analysis.*** The mRNA expression levels of PDLIM3 were examined in non-tumor brain samples, GBM and GSCs. Data for non-tumor brain, low grade gliomas and GBM samples were sourced from the TCGA, TCGA-GBMLGG, Rembrandt and CGGA databases, while the GSCs data were derived from RNA sequencing analyses previously conducted on GSCs obtained from 13 human GBM biopsy specimens. The RNA sequencing data utilized in this study are publicly accessible in the SRA database under the reference PRJNA1020743. All data were normalized against GAPDH expression. Kaplan-Meier survival analysis was conducted to assess the overall survival probabilities of patients with high versus low PDLIM3 expression. This analysis utilized data from the TCGA and CGGA databases via the Gliovis website (http://gliovis.bioinfo.cnio.es), with Wilcoxon tests applied in these evaluations. Differentially expressed genes between GSCs with high and low PDLIM3 levels were identified from the RNA sequencing dataset of the 13 patient samples by comparing the average expression of the PDLIM3-high group to that of the PDLIM3-low group, applying a fold change cutoff of ≥ 1.7 and a p-value of < 0.05. Gene ontology analysis on the significantly up-regulated genes in GSCs with elevated PDLIM3 expression was performed using the Metascape platform (https://metascape.org). To visualize the main pathways, an enrichment bubble plot was created using Srplot (http://www.bioinformatics.com.cn/srplot), an online tool for data analysis and visualization. The correlation between PDLIM3 expression in GSCs and invasion data was analyzed using Excel software. Pearson correlation coefficients and their corresponding p-values were calculated to assess the association between biomarkers and between biomarkers and invasion levels.

***3D tumor spheroid Invasion assay*** *.* The 3D tumor spheroid invasion assays were performed following the protocol outlined by Vinci M. et al. Briefly, cells were seeded into ultra-low attachment 96-wells round bottom plates to allow formation of a single spheroid/well. 72h later the spheroids were transfected or not with specific siRNA or a scramble. 24h after half of the growth medium was removed and replaced by Matrigel. After 1h at 37°C, when the Matrigel was solidified, growth medium was added to the wells. Images were captured at T0 and T24 hours with a Nikon microscope, using the Nikon software NIS Elements. The areas of the spheroids and the regions occupied by invading cells were measured using Image J software.

***SiRNA transfection, RNA extraction, Reverse Transcription, and Real-time PCR* .** Lipofectamine RNAi Max from Invitrogen was utilized for transfection in accordance with the manufacturer's instructions. After 48 hours, total RNA was extracted using the RNeasy Plus Micro Kit from Qiagen, followed by reverse transcription using the Prime Script RT Reagent kit from TAKARA. Real-time PCR was conducted with the ABI-Stepone+ system from Applied Biosystems, employing GAPDH for normalization. The siRNAs used in the experiment included si-PDLIM3(5), si-PDLIM3(6), and si-RNA negative control (SI03165715, SI04204564 and SI027310 from Qiagen, respectively).

***Neurosphere-forming analysis*** . Cells were transfected using designated siRNAs or a scrambled control and plated 48h later in 96-well plates at a density of 500 cells per well, with 12 wells allocated for each condition. 8-10 days after plating, the number of neurospheres per well (larger than 100 µM) was counted under a microscope.

***Statistical analysis.*** Unpaired T-tests were used to compare two groups. One-way Anova tests were performed for multiple comparisons. Wilcoxon tests were used in Kaplan-Meier survival analysis. For correlation analysis, Pearson correlation coefficients and their corresponding p-values were used.

## Reagents

**Table:** 

**Reagent**	**Source**
Dulbecco's Modified Eagle Medium (DMEM) F12	SIGMA D8437
N2 supplement	GIBCO (ThermoFisher Scientific) 17502-048
B27 supplement	GIBCO (ThermoFisher Scientific)17505-044
Human FGF-basic	Preprotech 100-18B
EGF	Preprotech AF-100-15
Matrigel Matrix	CORNING 354230
Lipofectamin RNAimax	ThermoFisher Scientific 13778150
RNeasy Plus Micro kit	QIAGEN 74034
Prime Script RT Reagent Kit	TAKARA
siRNA Hs_PDLIM3_5 SI03165715	QIAGEN
siRNA HS_PDLIM3_6 SI04204564	QIAGEN
si-RNA negative control SI027310	QIAGEN

## Data Availability

Description: Extended data 1: High expression of PDLIM3 and its correlation with patients survival as well as the expression of VIM, GPM6A and DBN1. Resource Type: Image. DOI: https://doi.org/10.22002/6sd82-07489 Description: Supplementary table S1: Genes significantly up-regulated in GSCs with a strong expression of PDLIM3 compared to GSCs expressing weakly PDLIM3 (fold change cutoff 1.7 pvalue ≤ 0.05) . Resource Type: Text. DOI: https://doi.org/10.22002/4be9r-g4h48
